# MERS Coronavirus: An Emerging Zoonotic Virus

**DOI:** 10.3390/v11070663

**Published:** 2019-07-19

**Authors:** Fang Li, Lanying Du

**Affiliations:** 1 Department of Veterinary and Biomedical Sciences, College of Veterinary Medicine, University of Minnesota, Saint Paul, MN 55108, USA; 2 Lindsley F. Kimball Research Institute, New York Blood Center, New York, NY 10065, USA

Middle East respiratory syndrome coronavirus (MERS-CoV) is an emerging virus that was first reported in humans in June 2012 [1]. To date, MERS-CoV continues to infect humans with a fatality rate of ~35%. At least 27 countries have reported human infections with MERS-CoV (https://www.who.int/emergencies/mers-cov/en/). MERS-CoV is a zoonotic virus. Like severe acute respiratory syndrome coronavirus (SARS-CoV), MERS-CoV is believed to have originated from bats [2,3]. However, whereas the bat-to-human transmission of SARS-CoV was likely mediated by palm civets as intermediate hosts, humans likely acquired MERS-CoV from dromedary camels [4,5,6]. Human-to-human transmission of MERS-CoV does occur, but it is limited mostly to health care environments [7,8]. Moreover, whereas SARS-CoV recognizes angiotensin-converting enzyme 2 (ACE2) as a cellular receptor [9,10], MERS-CoV uses dipeptidyl peptidase 4 (DPP4) to enter target cells [11,12]. Currently, no vaccines or antiviral therapeutics have been approved for the prevention or treatment of MERS-CoV infection, although a number of them have been developed preclinically and/or tested clinically [13,14,15,16].

The articles in this special issue of *Viruses* were written by researchers working in the MERS-CoV field. The main aims of this issue are to (i) better understand MERS-CoV transmission, epidemiology, and pathogenesis; (ii) summarize current progress on MERS-CoV animal models, vaccines, and therapeutics; and (iii) discuss future prospects for MERS-CoV research. This issue includes seven review articles and nine original research papers, each providing detailed updates on current MERS-CoV studies.

Studies on the transmission, epidemiology, and pathogenesis of MERS-CoV form one of the foundations of MERS-CoV research. In this issue, Farag and colleagues summarize the possible drivers of the emergence of MERS-CoV and its spillover to humans in Qatar, explaining the potential reasons for the camel-to-human transmission of MERS-CoV [17]. The review article by Song and colleagues provides an overall description of the epidemiology, pathogenesis, and other important aspects of MERS-CoV [18]. Widagdo and colleagues review the host determinants of the transmission and pathogenesis of MERS-CoV, indicating that receptor DPP4 plays an important role in these processes [19]. A research article by Yan and colleagues characterizes the role of lipid profiles in the pathogenesis and infectivity of human coronaviruses, including MERS-CoV, suggesting that lipid metabolism may be involved in the propagations of these coronaviruses [20]. These reports provide insights into how MERS-CoV infects cells and spreads within and across host species. They have also laid the foundations for developing animal models.

Animal models are essential tools for the preclinical evaluation of anti-MERS-CoV countermeasures. Dromedary camels, alpacas, and non-human primates are susceptible to MERS-CoV infection [21,22,23]; however, the virus does not infect small animals such as mice, hamsters, and ferrets [24,25,26]. Several mouse models that express human DPP4 (hDPP4) have been established for MERS-CoV infection [27,28,29]. In this issue, Widagdo and colleagues examine rabbits as potential hosts for MERS-CoV, showing that MERS-CoV infects rabbits without causing symptoms; they also analyze the route of MERS-CoV transmission in rabbits [30]. Fan and colleagues report the development of an hDPP4-expressing mouse model through inserting hDPP4 gene into a constitutive and ubiquitous gene expression locus using CRISPR/Cas9 technology. This mouse model is susceptible to MERS-CoV infection [31]. These articles have established platforms for testing vaccines and therapeutic agents targeting MERS-CoV.

Effective vaccines are essential for preventing MERS-CoV infection. The MERS-CoV surface spike (S) protein is a key target for vaccine design [14]. The S protein comprises two subunits: the S1 subunit is responsible for binding to the DPP4 receptor via a receptor-binding domain (RBD), and the S2 subunit mediates virus–host membrane fusion [32,33,34,35]. Several MERS-CoV S protein-based vaccines have been developed; when tested in animal models, they showed protective efficacy against MERS-CoV [14]. In this issue, Schindewolf and Menachery summarize the progress of MERS-CoV S-protein-based vaccine development and also describe potential challenges [36]. Zhou and colleagues review current advances in RBD-based MERS-CoV vaccines [37]. A research paper by Adney and colleagues evaluates the efficacy of a MERS-CoV S1 subunit vaccine aided by adjuvants; the authors report reduced and delayed viral shedding in dromedary camels as well as the complete protection of alpacas from MERS-CoV infection [38]. This and other studies demonstrate that the protective efficacy of MERS vaccines positively correlates with neutralizing antibody titers in serum [38,39]. In addition to inducing neutralizing antibodies, some types of vaccines can induce cellular immune responses against MERS-CoV. Other than the S protein, structural proteins such as the nucleocapsid (N) protein may also serve as vaccine targets. Here, Veit and colleagues report that a MERS-CoV N protein-based vaccine, which is delivered through a modified Vaccinia virus, induces CD8^+^ T cell responses in a mouse model; they further identify a MERS-CoV N protein-specific CD8^+^ T cell epitope on the vaccine [40]. Overall, these reports demonstrate that a variety of promising vaccine tools are available to prevent MERS-CoV infection in humans and other animals.

Therapeutics are critical tools for treating MERS-CoV infection. Again, the MERS-CoV S protein is an important target for therapeutic development [16]. MERS-CoV S2 contains two heptad repeat regions, HR1 and HR2, that are critical for S protein-mediated membrane fusion [34]. Hence, peptides mimicking HR1 or HR2 may interfere with the viral membrane-fusion process [34]. Moreover, RBD-targeting neutralizing monoclonal antibodies (mAbs) can block the viral attachment step [37]. In addition to conventional mAbs, single-domain antibodies isolated from camelids, called nanobodies (Nbs), can also block RBD/receptor interactions; these Nbs have been gaining popularity as therapeutic agents due to their small size and high stability [41,42]. Thus, both the HR1/HR2 peptide mimics and RBD-targeting mAbs and Nbs may serve as MERS-CoV entry inhibitors. Furthermore, small molecules targeting the S protein or nonstructural proteins may serve as therapeutic alternatives to peptide mimics and antibodies [16,43]. In this issue, two review articles report the current advances in therapeutic neutralizing antibodies, one by Han and colleagues and the other by Zhou and colleagues [37,44]. The latter article also discusses potential strategies and challenges to improving the efficacy of therapeutic neutralizing antibodies. In a research article, He and colleagues describe the construction and expression of dimeric and trimeric Nbs that target MERS-CoV RBD and further demonstrate the strong stability and high neutralizing activity of these Nbs against multiple MERS-CoV strains [42]. In another research article, Xia and colleagues report that three peptides mimicking HR2 from HKU4 (which is a MERS-related coronavirus from bats) strongly inhibit MERS-CoV infection [45]. Interestingly, Wang and colleagues report that the combination of a MERS-CoV HR2 peptide mimic and an RBD-targeting neutralizing mAb demonstrate potent synergistic effects in inhibiting MERS-CoV S protein-mediated viral entry [46]. In another research article, Jiang and colleagues report that an antibody targeting complement receptor C5aR1 inhibits MERS-CoV infection, indicating that MERS-CoV infection elicits the over-activation of the complement system, and this process can be blocked by anti-C5aR1 antibodies [47]. Moreover, Liang and colleagues review advances in the development of small-molecular MERS-CoV inhibitors [48]. Overall, these articles confirm that anti-MERS-CoV therapeutics have great potentials in treating MERS-CoV infections in humans and other animals. 

To summarize, significant progress has been made in MERS-CoV research in the past seven years since the virus was discovered. This progress includes, but is not limited to, the epidemiology, transmission, and pathogenesis of MERS-CoV, as well as animal models, vaccines, and antivirals for MERS-CoV. This special issue of *Viruses* provides updated reports on this progress. However, challenges remain. For example, we still do not understand how exactly MERS-CoV transmits from bats to camels or humans. Moreover, compared to HIV and influenza viruses, the potential market for MERS-CoV vaccines and therapeutics is much smaller, making commercialization of MERS-related products more challenging. Nevertheless, the past two decades have witnessed the emergence of two highly pathogenic coronaviruses, MERS-CoV and SARS-CoV. While these two viruses remain significant threats to global health, future novel coronaviruses with pandemic potential may emerge from their animal reservoirs and infect humans. Thus, research into MERS-CoV should remain a high priority for the virology community. In fact, the impressive progress in MERS-CoV research has benefitted tremendously from previous research into coronaviruses including SARS-CoV. Therefore, scientists’ current efforts regarding MERS-CoV will prepare humans to battle any future novel coronaviruses with pandemic potential.
